# Novel Combination of Lateral Interbody Fusion and Endoscopic Ipsi-Contra Decompression for Severe Stenosis From Lumbar Spondylolisthesis: A Case Report

**DOI:** 10.7759/cureus.60160

**Published:** 2024-05-12

**Authors:** Sumedh S Shah, Malek Bashti, Manav Daftari, Gregory W Basil

**Affiliations:** 1 Neurological Surgery, University of Miami Miller School of Medicine, Miami, USA

**Keywords:** spine, lateral approach, minimally invasive spine surgery, fusion, endoscopy

## Abstract

Minimally invasive surgical approaches to the spine that leverage indirect decompression are gaining increasing popularity. While there is excellent literature on the value of indirect decompression, there are limitations to this procedure. Specifically, in patients with severe stenosis and neurogenic claudication, there is a concern among many surgeons regarding the adequacy of indirect decompression alone. In these cases, the lateral approach is often abandoned in favor of an open posterior or posterior minimally invasive approach. Unfortunately, some of the distinct benefits of the direct lateral approach are then lost. Here, we present the case of a 58-year-old male who underwent an L4-L5 lateral interbody fusion with an endoscopic ipsi-contra decompression to achieve both direct and indirect treatment of severe neuroforaminal and central stenosis. From this strategy, this patient had complete pre-operative symptom resolution and was able to return to work immediately after surgery without significant restriction. Combining the benefits of direct and indirect using an ultra-minimally invasive decompressive approach offers a potential solution.

## Introduction

Symptomatic lumbar stenosis significantly impacts health, mobility, and quality of life, leading to high social and healthcare cost. Traditional treatment for this pathology is typically direct open decompression with midline laminectomy. In some cases, this decompression may be combined with fusion surgery depending on patient pathology and symptoms. These methods, however, entail considerable disruption of normal anatomy leading to long-term sequelae [1]. Open surgery also increases pain, blood-loss, operative time, and prolonged recovery [2,3]. As a result of these complications, a number of minimally invasive surgical (MIS) approaches to the spine have been developed. One approach, the direct lateral lumbar interbody fusion (DLIF) achieves indirect decompression via ligamentotaxis, reduces morbidity and improves recovery. However, lateral approach effectiveness varies with factors like spondylolisthesis grade, age, anatomy, and faces limitation in patients with severe radicular symptoms at rest [4,5]. Literature suggests that indirect decompression with pedicle screw fixation can increase vertebral canal cross-sectional area, yet patients with severe central stenosis may require a more robust direct decompression [6,7].

Recent studies suggest that while both direct and indirect offer symptom relief, the degree of improvement is better in direct decompression and re-operation rates may be higher in indirect decompression [8]. Direct decompression can be achieved in an MIS fashion, such as with endoscopic approaches, however, the combination strategies are not well reported. Therefore, an optimal treatment strategy would seek to combine the benefits of MIS indirect approaches with the durability of MIS direct posterior decompression. This case report presents a novel approach combining lateral lumbar fusion with endoscopic ipsi-contra decompression, addressing traditional method limitations while minimizing invasiveness. It marks a safe and novel treatment paradigm for severe lumbar stenosis with axial back pain and spondylolisthesis.

## Case presentation

Pre-operative course

**Figure 1:**
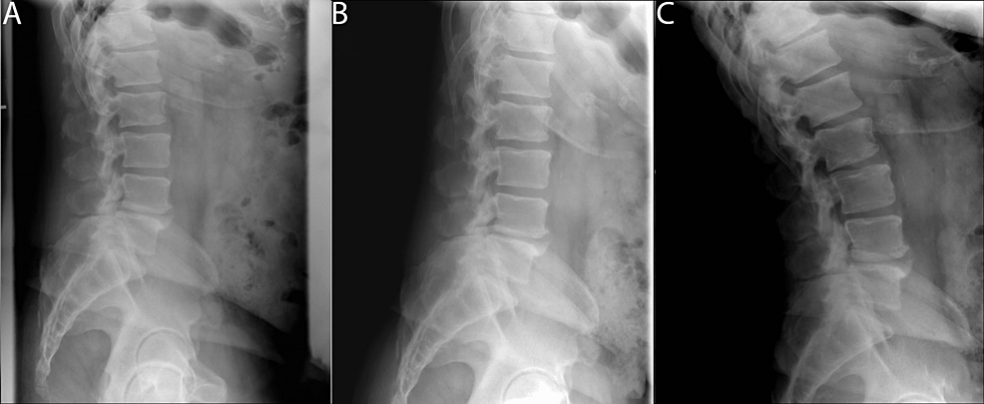
Pre-operative dynamic X-ray images of the lumbar spine showing grade 1 spondylolisthesis of L4-L5 (A) neutral (B) flexion (C) extension.

**Figure 2:**
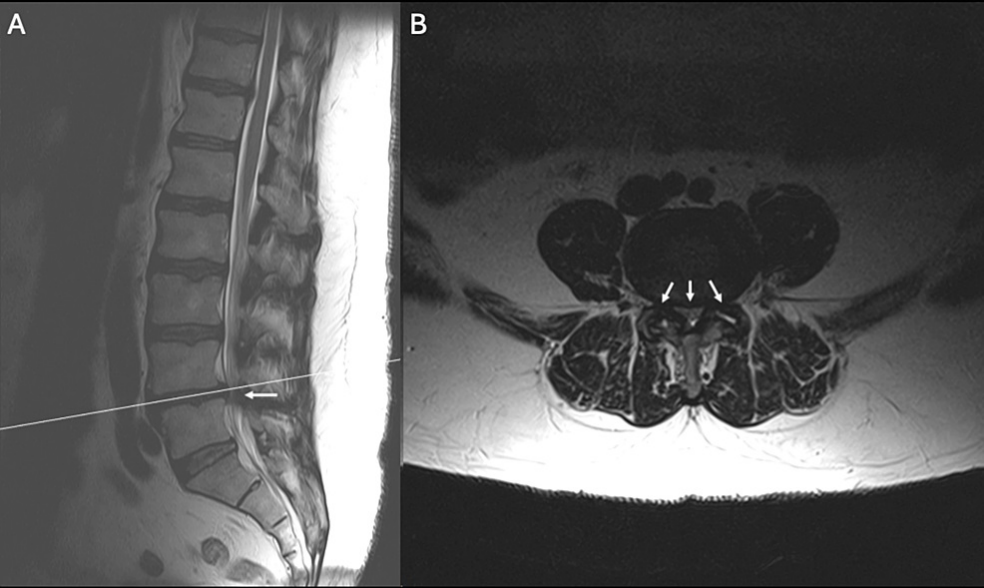
Pre-operative MRI scans of the lumbar spine in sagittal (A) and axial (B) planes demonstrating grade one spondylolisthesis at L4-L5 with notable ligamentum hypertrophy and severe central and arrows marking bilateral neuroforaminal stenosis, as indicated by arrows.

A 58-year-old male with hypertension and diabetes presented with severe lower back pain and neurogenic claudication. At the time of his presentation, he reported unrelenting bilateral leg pain even at rest. His symptoms progressed over five weeks and were refractory to conservative treatments including physical therapy, epidural injections, and oral medications. Imaging was performed and demonstrated a grade 1 spondylolisthesis at L4-L5 accompanied by significant ligamentum hypertrophy leading to severe central and bilateral neuroforaminal stenosis (Figures 1, 2). Severe central stenosis was evaluated via the Schizas et al. grading system showing compression of the rootlets with effacement of the cerebrospinal fluid space but showing epidural fat [9].

Given the patient's clinical presentation, the lack of response to conservative measures, and the pre-operative imaging findings, a decision was made to pursue a minimally invasive surgical approach. We opted to combine lateral fusion with endoscopic ipsi-contra ligamentum flavum resection to achieve combined direct and indirect decompression during a single surgical session. The patient consented to the procedure.

Surgical technique

The procedure commenced with fluoroscopy guided optimization of patient positioning for the lumbar interbody fusion (LIF) approach. Precise incisions and careful dissection allowed access to the L4-L5 disc space. Dissection was performed through the external and internal oblique muscles, transversalis fascia, and along the retroperitoneum until the psoas muscle was visualized. The psoas muscle was dilated using directional evoked electromyography (EMG). The dilator was taken down to the disc space. Progressive dilation was performed followed by the placement of a retractor opened up, down, and posteriorly. Following annulotomy and disc release, a 3D printed titanium cage, NuVasive Inc., San Diego CA, USA, augmented with bone morphogenetic protein and allograft cadaveric bone, was positioned under fluoroscopic guidance. The integrity and correct placement of the implant were verified. The incision was irrigated and closed in the usual fashion.

The patient was repositioned prone for the endoscopic ipsi-contra decompression and prepped in the usual sterile fashion. The midline was localized using anteroposterior (AP) fluoroscopy, and an incision near the midline (~1.5 cm paramedian) facilitated access to the L4 lamina via the working channel and endoscope. Careful removal of bony structures and ipsilateral and contralateral portions of the ligamentum flavum was performed using endoscopic Shrill with a diamond tip, Kerrison rongeurs, and endoscopic bipolar electrocautery. Adequacy of decompression was determined under direct visualization.

The surgery concluded with the placement of percutaneous screws. There were no operative complications, and the estimated blood loss was < 50 mL. No changes in intraoperative monitoring were noted.

Post-operative course and follow-up

**Figure 3:**
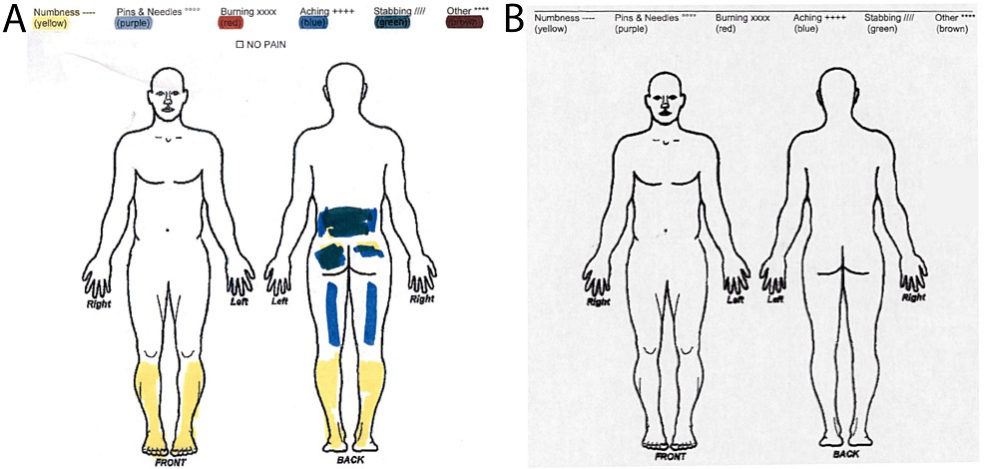
Patient's pain diagram indicating pre-operative symptoms with areas of numbness (yellow) in both legs, pins, and needles (purple) radiating down the left leg (A). The absence of any pain indicators five weeks post-operative indicates pre-operative pain relief (B).

Postoperatively, the patient experienced rapid recovery, was discharged on day three, and returned to work on day six; demonstrating significant pain improvement at a two-week follow-up and complete symptom resolution by six weeks (Figure 3).

**Figure 4:**
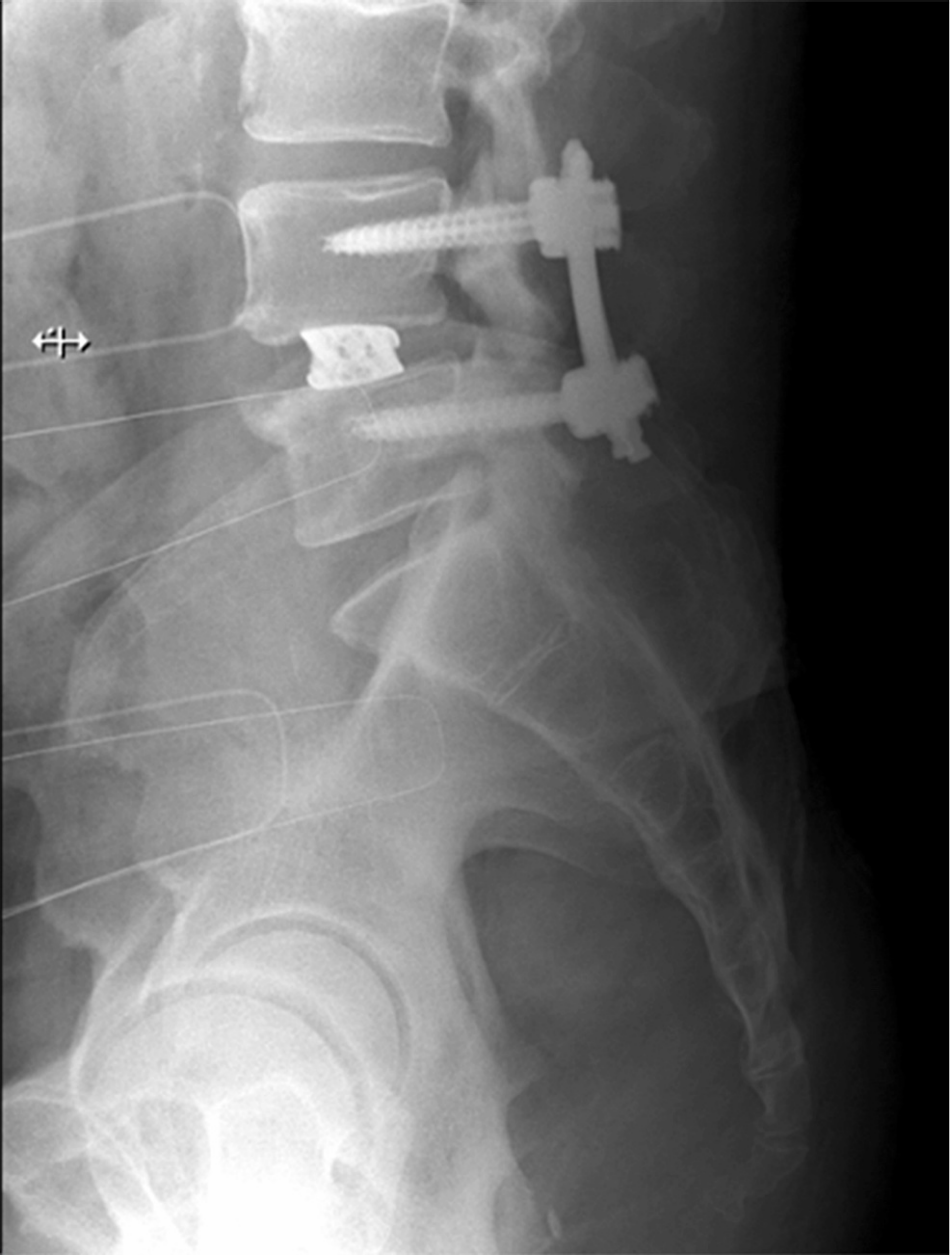
Post-operative lateral lumbar spine X-ray showing successful placement of instrumentation after L4-L5 lateral interbody fusion with endoscopic ipsi-contra decompression, indicating proper alignment and stability with no signs of complication.

Symptomatic resolution was sustained throughout the five-month post-operative period. Post-operative imaging confirmed appropriate instrument placement (Figure 4).

## Discussion

Lumbar spondylolisthesis and canal stenosis have been historically managed by open decompressive and fusion techniques. However, emerging evidence suggests that MIS procedures offer significant advantages. Studies show open approaches increase length of stay, and post-operative pain, and decrease patient satisfaction [10]. Conversely, minimally invasive procedures have reduced pre-operative pain and improved outcome metrics [11].

The DLIF technique is linked to long-term clinical improvement, fewer complications, and enhanced patient satisfaction one year post-operatively compared to direct decompression [12,13]. However, in cases of severe degenerative stenosis indirect decompression often requires subsequent direct decompression, as shown in McKeithan et al.'s study of 4145 patients, where indirect decompression led to higher reoperation rates and lower satisfaction [8]. This is relevant in severe central spinal stenosis, where indirect decompression alone often necessitates revision direct decompression surgery [14,15]. Direct open decompression, while effective, carries risks of complications including spinal cord injury, epidural hematoma, and iatrogenic injury [1,10,16]. Therefore, MIS direct decompression approaches, including endoscopic options, are becoming more commonplace to treat posterior compression.

Here, we opted to combine the lateral fusion approach with endoscopic ipsi-contra decompression. This unique combination restored physiologic alignment and disc height and alleviated back pain, claudication, leg pain, and numbness associated with central canal stenosis without significant disruption of anatomy.

Complementing LIF with endoscopic ipsi-contra decompression captured the benefits of both lateral fusion and endoscopic decompression approaches. Notably, the lateral approach permits a large interbody cage placement with good endplate contact, a concurrent higher fusion rate, lower infection rate, and decreased disruption of paraspinal tissue [17,18]. Endoscopic central decompression also offers decreased postoperative pain, minimal tissue disruption, and early return to normal activity [19]. This synergy potentially offers a more comprehensive decompression, addressing both central and foraminal stenosis, and is particularly advantageous in patients with complex spinal pathology as demonstrated in this case. The patient's rapid recovery and significant pain relief underscore this combined approach’s efficacy. Minimal blood loss, short hospital stay, and early return to work highlight the advantages of MIS techniques. These outcomes align with the growing body of evidence suggesting that minimally invasive spine surgery can reduce perioperative morbidity and facilitate quicker recovery compared to traditional open surgery. To our knowledge, this is the first instance of combining these two approaches in a single surgical setting. Additional research with larger case numbers will ultimately be necessary to show consistently favorable outcomes, however, we demonstrated a combination approach can be done for patients requiring complex treatments safely and with acceptable outcomes.

## Conclusions

This successful application of a combined lateral and endoscopic ipsi-contra decompression technique offers a promising strategy to treat severe symptomatic central stenosis. It combines an MIS direct and indirect approach, potentially minimizing the need for revision surgery while reducing the risk of the complications typically associated with open methods. It will be imperative to conduct further research, including larger cohort studies and long-term follow-up, to validate these findings and establish a more comprehensive understanding of the benefits and risks involved in this novel surgical approach.
